# 93. Novel recombinant measles virus vaccine prevents measles virus disease in rhesus macaques

**DOI:** 10.1093/ofid/ofac492.018

**Published:** 2022-12-15

**Authors:** Jessica Rubens, Jacqueline Brockhurst, Shristi Ghimire, Rebecca Loomis, Alexandrine Derrien-Colemyn, Jason Villano, Guillaume Stewart-Jones, Tracy Ruckwardt, Michael Watson, Barney S Graham, Diane Griffin

**Affiliations:** Johns Hopkins University School of Medicine, Baltimore, Maryland; Johns Hopkins University School of Medicine, Baltimore, Maryland; Johns Hopkins Bloomberg School of Public Health, Baltimore, Maryland; NIH/VRC, Bethesda, Maryland; NIAID/NIH, Bethesda, Maryland; Johns Hopkins Medical Institutions, Baltimore, Maryland; MeVox, Oxford, England, United Kingdom; NIH/VRC, Bethesda, Maryland; Mevox Ltd, Oxford, England, United Kingdom; Morehouse School of Medicine, Smyrna, Georgia; Johns Hopkins Bloomberg School of Public Health, Baltimore, Maryland

## Abstract

**Background:**

Measles-Mumps-Rubella (MMR) is an effective live-virus vaccine against measles virus (MeV). However, use of MMR is limited by its inability to boost MeV immunity, lack of immunogenicity in infants, and contraindication in pregnant and immunocompromised persons.

**Methods:**

We evaluated a novel recombinant dimeric MeV hemagglutinin protein vaccine (rMeV) in a rhesus macaque model. Sixteen macaques were primed at day 0 and boosted at day 42 by experimental group: 1) MMR x2; 2) rMeV x2; 3) MMR prime/rMeV boost; 4) control; n=4. Macaques were challenged intratracheally with Bilthoven strain wild type MeV 8 months later. Blood, bone marrow (BM), and lymph node (LN) samples were collected over 3–28 days after challenge. Replication-competent MeV was measured in peripheral blood mononuclear cells (PBMC), BM cells, and LN cells by infectious assay; MeV RNA in PBMC and BM cells was determined by quantitative reverse transcriptase polymerase chain reaction. Plasma was evaluated for MeV-specific IgG and plaque reduction neutralization titer (PRNT).

**Results:**

Six months after vaccination, mean PRNT was 2,432 in rMeV x2 (standard deviation (SD) 3,840), 3,584 in MMR-prime/rMeV boost (SD 3,072) and 5,120 in MMR x2 groups (SD 3,547). Upon infectious challenge, macaques who received any MeV-containing vaccine developed no clinical signs of measles and had no detectable infectious virus in PBMC, BM cells, or LN cells. All unvaccinated macaques had virus in PBMC that peaked at day 7 (mean 3,162 TCID50/mL, SD 4.1) and resolved by day 14 post challenge, and one macaque developed an extensive rash. Macaques who received any MeV-containing vaccine had no detectable MeV RNA in PBMC or BM cells, whereas all unvaccinated macaques had detectable MeV RNA that peaked at day 7 (1.6e5 copies, SD 10.5) in PBMC. In all experimental groups, MeV-specific IgG titers increased after MeV challenge.

**Conclusion:**

Macaques who received rMeV and/or MMR were protected from rash, viremia, and detection of MeV RNA in PBMC and BM cells, unlike unvaccinated macaques. These data suggest that rMeV vaccine generates protective immune responses against measles and may be a novel candidate for future measles vaccine strategies. Study of cellular responses after rMeV vaccination and MeV challenge is warranted.

**Disclosures:**

**Jessica Rubens, MD**, Mevox: Grant/Research Support **Guillaume Stewart-Jones, PhD**, Moderna: Inventor of SARS-CoV-2 vaccine sequences|Moderna: Stocks/Bonds **Michael Watson, MD**, MEVOX Ltd: Board Member|MEVOX Ltd: Ownership Interest|MEVOX Ltd: Stocks/Bonds **Barney S. Graham, MD, PhD**, BSG: BSG is an inventor on patents for the stabilization of the RSV F protein (WO2014160463A1, Prefusion RSV F proteins and their use).|National Institutes of Health: Inventor on patents for RSV vaccines|National Institutes of Health: inventor on patents for measles and other paramyxovirus vaccines **Diane Griffin, MD PhD**, Gilead: Grant/Research Support|GlaxoSmithKline: Advisor/Consultant|GreenLight Biosciences: Advisor/Consultant|Merck: Advisor/Consultant|MeVox: Grant/Research Support|Takeda Pharmaceuticals: Advisor/Consultant.

